# Geometric Analyses of the Expiratory Flow–Volume Curve to Identify Expiratory Flow Limitation During Exercise

**DOI:** 10.3390/fluids10040094

**Published:** 2025-04-03

**Authors:** Hans Haverkamp, Gregory Petrics, Yannick Molgat-Seon

**Affiliations:** 1 Department of Nutrition and Exercise Physiology, Elson S. Floyd College of Medicine, Washington State University, 412 E. Spokane Falls Blvd., Spokane, WA 99202, USA; 2 Department of Mathematics, Vermont State University, Johnson, VT 05656, USA;; 3 Department of Kinesiology and Applied Health, University of Winnipeg, Winnipeg, MB R3B 2E9, Canada;

**Keywords:** breathing mechanics, cardiopulmonary exercise testing, deep learning, dyspnea, exercise, exertional dyspnea, ventilatory constraint

## Abstract

An important purpose of cardiopulmonary exercise testing (CPET) is to query the mechanisms for unexplained shortness of breath or exaggerated exertional dyspnea. Expiratory flow limitation (EFL) is an important indicator of ventilatory constraint that can negatively influence both dyspnea and exercise capacity. Unfortunately, due to logistical challenges and lack of sufficient clinical training, EFL is rarely measured during CPET. The conventional method for identifying exercise EFL is limited because it requires patient cooperation and it is also dependent on the maximal expiratory flow–volume curve, which underestimates actual maximal expiratory flow during exercise. Simplified methods for identifying EFL that are based on the shape of the exercise tidal flow–volume curve would improve the accessibility of measuring EFL during exercise. The overall aim of this review is to critically review the approaches and methods used to measure EFL in exercising adults. We review the physiology underlying EFL and the conventional methods for determining exercise EFL. We then provide critical analyses of more recent methods for identifying exercise EFL that are based on the geometry of the exercise tidal expiratory flow–volume curve. Finally, we highlight recent work designed to assess exercise EFL using a type of deep machine learning known as a convolutional neural network.

## 1. Introduction

During whole-body exercise, alveolar ventilation must increase in proportion to metabolic rate in order for arterial blood oxygen content—and thus oxygen transport—to be preserved. In healthy adults with normal to above-average aerobic capacity, the structural and functional characteristics of the pulmonary system are overbuilt; the system accommodates the required increases in inspiratory and expiratory airflow even during maximal exercise. However, in well-trained aerobic athletes and in adults with pulmonary disease, exercise expired airflow can reach the maximum flow permissible by the structural and functional characteristics of the airways and lung parenchyma. Such expiratory flow limitation (EFL) contributes to exercise limitation and exertional dyspnea (i.e., shortness of breath) in healthy adults and persons with pulmonary disease [1–5].

An important purpose of cardiopulmonary exercise testing (CPET) is to query the mechanisms for unexplained shortness of breath or exaggerated exertional dyspnea. Despite the insights provided by knowledge of the presence of exercise EFL, it is rarely measured during CPET. There are several reasons for this, including lack of sufficient clinical training combined with limitations of CPET systems, among others (discussed below). In response to these limitations, several methods for identifying exercise EFL have been developed that are based on the shape of the spontaneous exercise tidal expiratory flow–volume (TEFV) curve rather than the conventional approach of assessing EFL that involves placing the TEFV curve within the maximal expiratory flow–volume (MEFV) curve. These approaches have failed to gain traction in research or clinical spaces. Despite this, there is a sound rationale supporting the coupling of TEFV curve configuration with the presence of EFL during exercise [6]. Given the limitations of the conventional approach for measuring EFL, pursuit of novel methods for its identification are warranted.

This manuscript begins with an overview of exercise EFL, including a description of its physiology, a review of the most employed methods for its measurement, and a discussion of the physiological consequences of EFL. Then, we provide a critical review of several proposed approaches for identifying EFL that are based on the configuration of the TEFV curve. Lastly, the largely unexplored, yet promising, potential of deep machine learning for identifying EFL is highlighted and discussed.

## 2. What Is Expiratory Flow Limitation?

During a forceful expiration, flow initially increases as a function of transpulmonary pressure (i.e., the difference between airway pressure and pleural pressure); however, at any given lung volume, there is a point where expiratory flow reaches a mechanical limit and cannot be increased further. This mechanical event has been termed EFL, and it can be defined as a situation where expiratory flow ceases to increase even with increasing expiratory effort [7]. In order to understand EFL and its physiological mechanisms, it is essential to recognize the factors that determine the maximal capacity to generate expiratory flow and the extent to which ventilatory output encroaches on the maximal capacity to generate expired flow at rest and during exercise across the continuum of health and disease.

**Figure 1.**
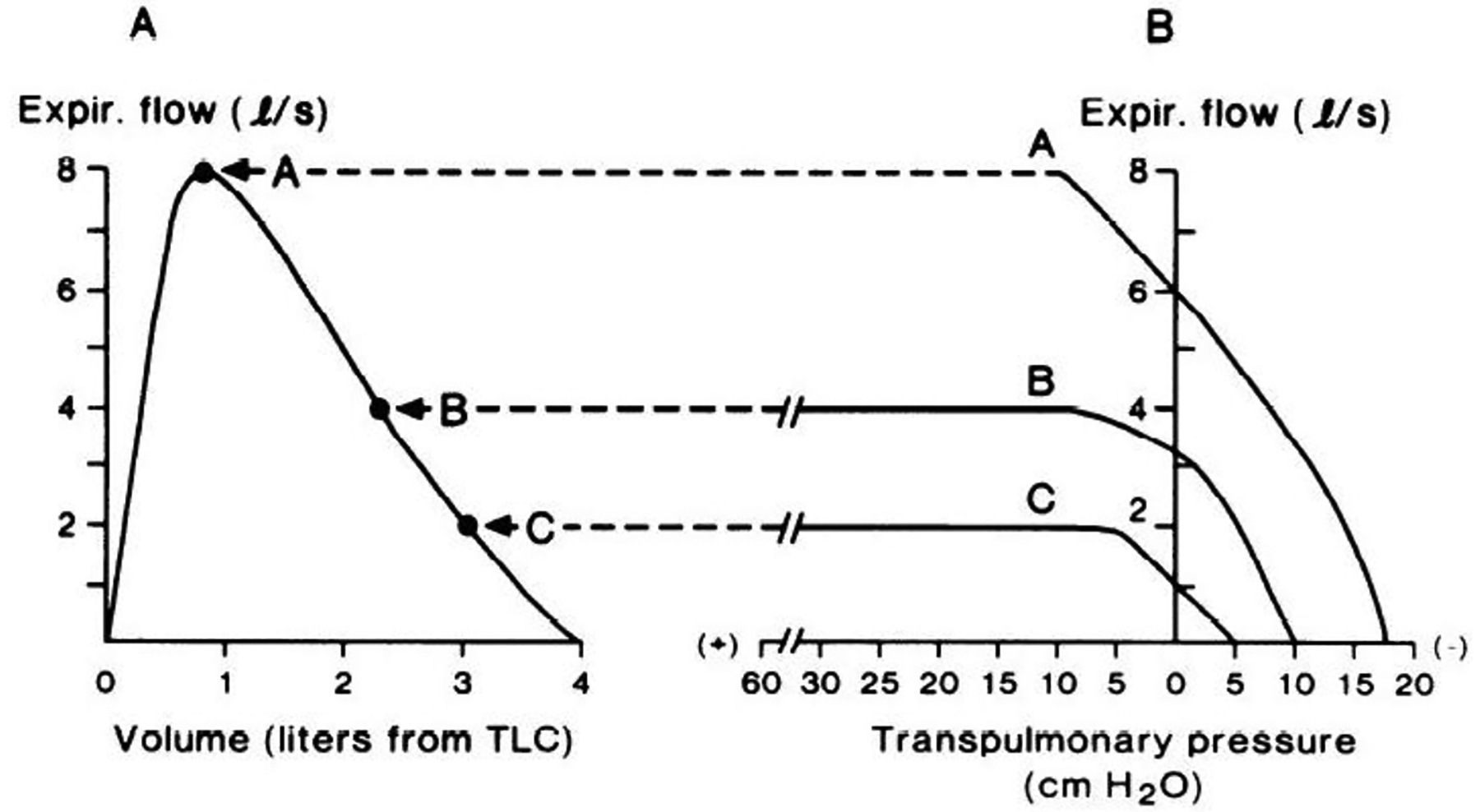
(**A**) Maximal expiratory flow–volume curve from total lung capacity to residual volume. Expiratory flow increases rapidly to a peak value early during expiration. As expiration continues, flow decreases in concert with smaller lung volumes. (**B**) Expired airflow (ordinate) plotted as a function of transpulmonary pressure (abscissa) at three lung volumes. At the high lung volume (curve A), airflow continually increases with increasing pressure. However, at the lower lung volumes (curves B and C), expiratory flow plateaus despite progressive increases in transpulmonary pressure. Thus, expiratory flow becomes “flow-limited” and independent of effort. Reproduced with permission from Hyatt R.E., *J Appl Physiol Respir Environ Exerc Physiol*; 1983 55(1 pt 1):1–7.

The human respiratory system has a finite capacity to generate flow. Inspiration is an active process, whereby inspiratory flow results from the negative transpulmonary pressure that is generated by the contraction of the inspiratory musculature [8]. Inspiratory flow can be increased with increasing force output from the inspiratory muscles, and the maximal capacity for inspiratory flow generation is essentially proportional to inspiratory muscle strength [9,10]. Thus, inspiratory flow across the range of lung volume from residual volume to total lung capacity is said to be ‘effort-dependent’. At rest, expiration is a passive process, whereby expiratory flow is generated by the passive recoil of the respiratory system. However, when expiratory flow must be increased (e.g., exercise hyperpnea, coughing, etc.), expiration becomes an active process, involving contraction of the expiratory muscles and a corresponding increase in transpulmonary pressure. Under such conditions (i.e., forced expiration), if expiratory effort increases above a certain level, expiratory flow is limited by the mechanical properties of the airways rather than by expiratory muscle force output. Indeed, the relationship between transpulmonary pressure and maximal expiratory flow is not linear, and varies depending on lung volume, as shown in Figure 1B. When considering a forced expiratory maneuver from total lung capacity to residual volume, the early phase of expiration (i.e., from total lung capacity to ~80% of vital capacity, indicated by curve A in Figure 1B) has been described as being ‘effort-dependent’, since expired flow is determined by expiratory muscle force output; however, experimental evidence suggests that even at lung volumes close to total lung capacity, expiratory flow can still become limited [11]. At lower lung volumes (curves B and C in Figure 1B), maximal expiratory flow is relatively ‘effort-independent’ and determined by the physical properties of the airways and lungs rather than by expiratory muscle force and transpulmonary pressure [12,13].

According to the concept of the ‘equal pressure point’, maximal expiratory flow is proportional to the elastic recoil pressure of the lungs and inversely proportional to airway resistance, whereby expiratory flow becomes limited when transmural pressure (i.e., the difference between alveolar pressure and plural pressure) is zero [14]. Given that the elastic recoil pressure of the lungs falls with decreasing lung volume [14], maximal expiratory flow follows the same trajectory and forms the border of the MEFV curve (Figure 1A), thereby describing, in visual and quantitative terms, the maximum capacity for expiratory flow generation.

In the context of EFL, ventilatory output refers to the amount of airflow, and by association volume, exhaled from the airways and lungs over a given period of time, which can be represented graphically by a TEFV curve. The configuration of the TEFV essentially reflects minute ventilation (i.e., the product of tidal volume and breathing frequency). Minute ventilation is tightly linked to whole-body metabolism, whereby it is regulated to ensure adequate diffusion of oxygen in to, and carbon dioxide out of, the blood such that arterial blood gas homeostasis is maintained, even under conditions of physiological stress [15]. For example, during aerobic exercise, skeletal muscle metabolism increases oxygen consumption and carbon dioxide production, which leads to a corresponding increase in minute ventilation. When ventilatory output increases, the associated increase in expiratory flow encroaches on the respiratory system’s finite ability to generate expired flow and can, in some cases, lead to EFL. Thus, the configuration of the TEFV curve, and its location within the boundary of the MEFV curve, is central to the assessment of EFL [16].

Overall, EFL occurs when ventilatory output meets the respiratory system’s maximal capacity to generate expiratory flow, which may result from the situation where ventilatory output is high, the maximal capacity to generate expired flow is low, or both. Additionally, EFL occurs in some, but not all, individuals, and its frequency and severity vary considerably in the general population. Healthy young adults (i.e., aged 20–30 years) have a remarkable, generally overbuilt, capacity to generate expired flow, which implies that EFL only occurs when ventilatory output is increased well above resting levels, as is the case during high-intensity aerobic exercise in well-trained persons with high peak aerobic power (i.e., >65 mL·kg^−1^·min^−1^) and correspondingly high ventilatory demand [17]. In fact, EFL only occurs in ~50% of healthy adults during exercise, usually at or near physiological maxima [18]. Although there is evidence that females are more likely to experience EFL during exercise than their male counterparts (40), presumably due to sex differences in respiratory system morphology, this is only the case in highly-trained endurance athletes (i.e., average peak aerobic power of 70 and 60 mL·kg^−1^·min^−1^ in males and females, respectively) and healthy older adults (i.e., aged ≥60 years) (40). Indeed, recent reports indicate that sex differences in the propensity towards EFL during exercise are not present in healthy young individuals of average aerobic fitness (18). In healthy older adults, the age-related decline in elastic recoil pressure of the lungs decreases the ability to generate expired flow [19]. Moreover, aging causes impairments in the distribution of ventilation that increase the ratio of dead space-to-tidal volume, thereby increasing the ventilatory output needed to maintain blood-gas homeostasis at a given metabolic rate [20]. Thus, older adults are more prone to exercise-induced EFL than healthy younger adults [21]. Moreover, the increased likelihood of experiencing EFL in older adults than younger adults is seen at both lower absolute and lower relative workloads and minute ventilation (21). In patients with chronic airway obstruction, such as chronic obstructive pulmonary disease (COPD) or asthma, pathological reductions in both the ability to generate expired flow and ventilatory efficiency further increase the likelihood of developing EFL both at rest [22] and during exercise [2,23,24] when compared to their healthy age-matched counterparts.

There are several methods to assess EFL in spontaneously breathing humans at rest and during exercise, and each of these methods have unique benefits and drawbacks that affect the validity of the associated measures and the quality of the data derived therefrom [25,26]. A comprehensive summary of all the methods used to assess EFL is beyond the scope of this review. Herein, we focus on the ‘gold-standard’ method of assessing EFL (i.e., iso-volume pressure–flow curves) and the conventional (yet still contemporary) approaches based on measures of flow and volume (i.e., flow–volume curves), as well as how these approaches may inform the development and implementation of geometric methods of identifying EFL.

### 2.1. Iso-Volume Pressure–Flow Curves

By definition, EFL occurs when increases in transpulmonary pressure do not result in further increases in expiratory flow [7]. The maximum capacity to generate expiratory flow varies proportionately with lung volume [14]. Thus, the most direct method to assess EFL would, at the very least, require simultaneous measures of expiratory flow, lung volume, and transpulmonary pressure. However, during tidal breathing, as lung volume changes, so too does the caliber of the airways [27], which complicates the assessment of EFL. Fry et al. [28] developed a method that describes the aerodynamic behavior of the lung at a fixed lung volume that can be used to define the exact transpulmonary pressure at which expiratory flow plateaus and becomes limited (Figure 1B). In essence, this is a direct measure of EFL; however, this technique is methodologically challenging. Participants must be sealed within a bespoke whole-body plethysmograph, estimates of pleural pressure must be obtained using intra-esophageal manometry, and measures must be performed at several lung volumes in order to characterize the range of effective transpulmonary pressures from total lung capacity to residual volume. Perhaps unsurprisingly, this method is primarily used in research settings to understand the mechanisms of maximal expiratory flow and how they are affected by aging and respiratory disease, and cannot feasibly be applied to standard CPET.

### 2.2. Flow–Volume Curves

**Figure 2.**
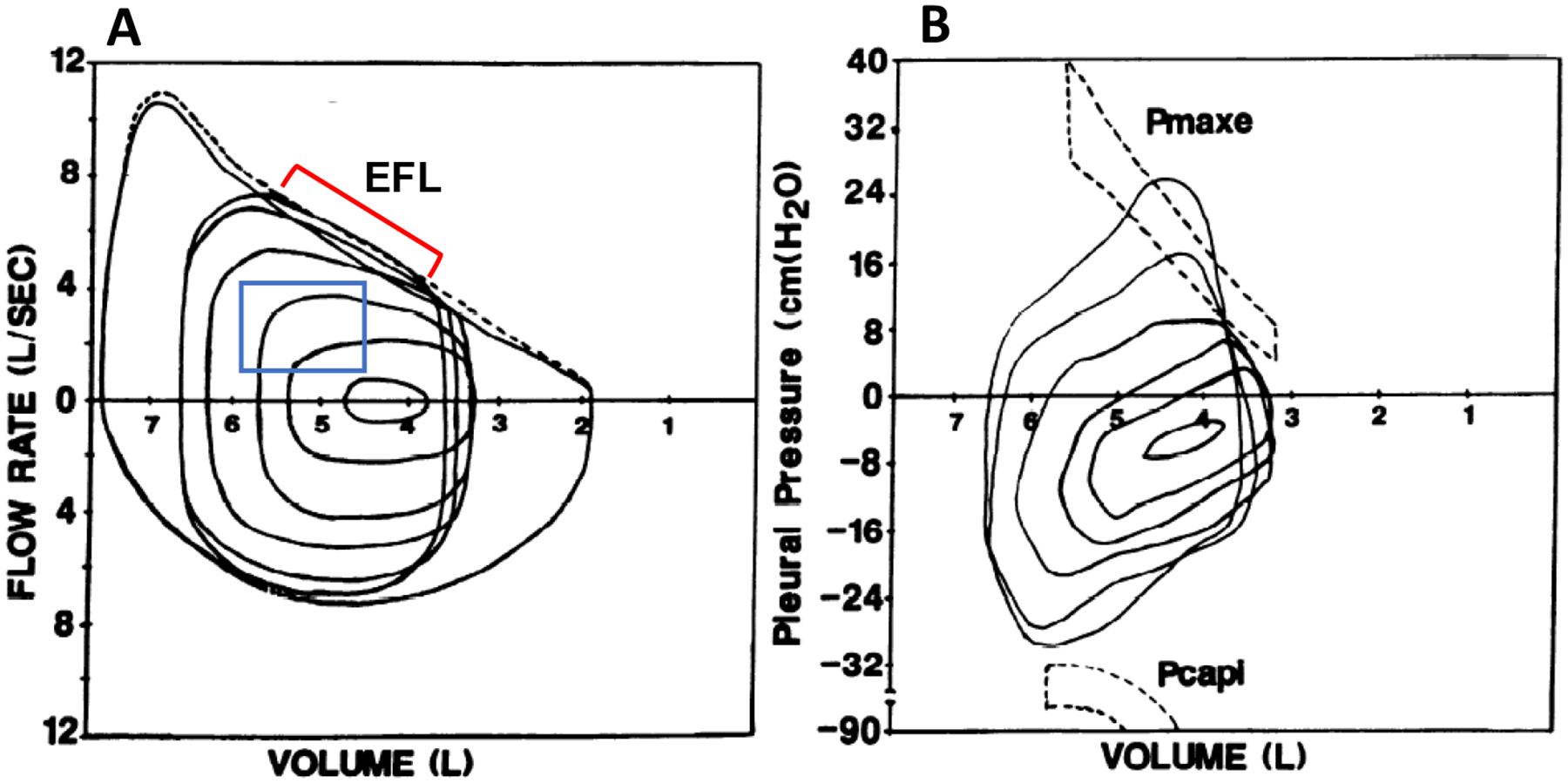
(**A**) Maximal volitional flow–volume loops with one resting and five spontaneous exercise tidal flow–volume loops plotted within the maximal loops. The exercise breaths contained within the blue box did not intersect the maximal flow–volume loops and are not expiratory flow-limited. The breaths indicated by the red bracket do intersect the maximal flow–volume loops and are thus expiratory flow-limited. (**B**) Pressure–volume loops at rest and during exercise in the same subject. Maximal effective expiratory pressures (Pmaxe) are contained within the dashed lines in the upper half of the figure. Note that Pmaxe decreases at lower lung volumes. Pcapi, maximum inspiratory pressure at a given lung volume and flow rate. Adapted with permission from [17]

**Figure 3.**
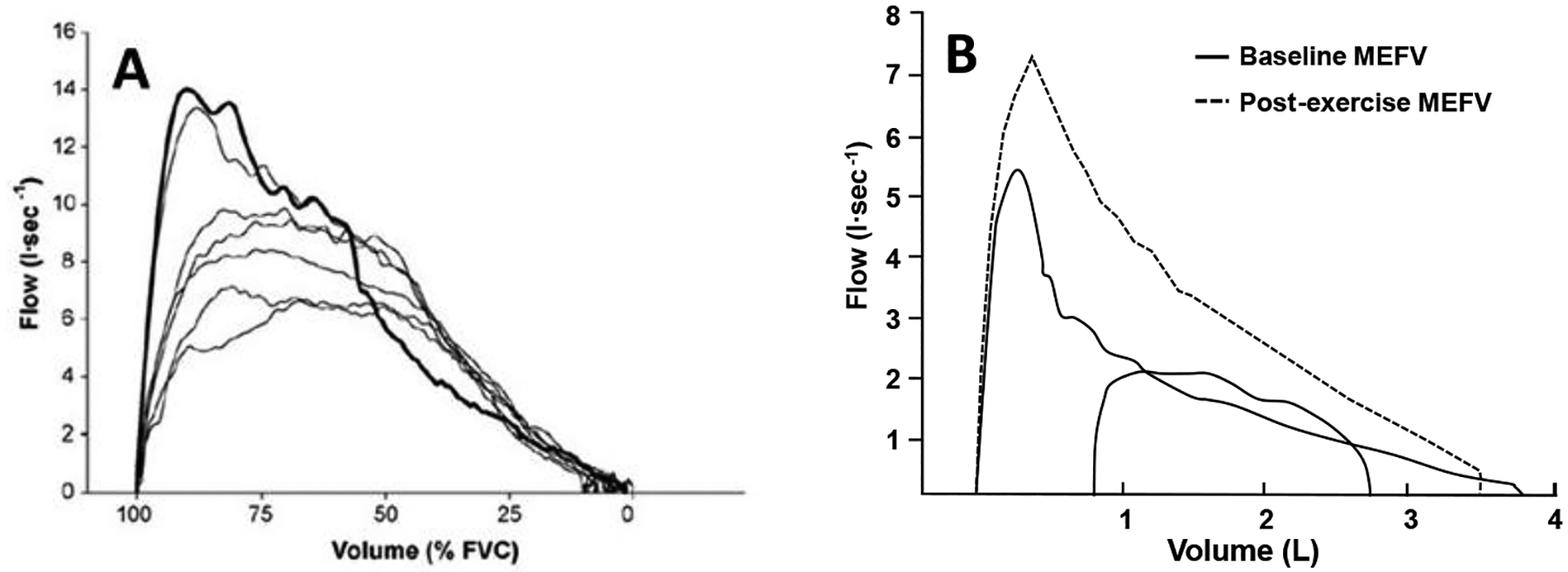
(**A**) Maximal forced expiration (bold line) and several expirations at varying efforts from total lung capacity in a healthy adult. The higher expiratory airflows during the graded efforts are due to reductions in thoracic gas compression at the reduced transpulmonary pressures. This method can be used to account for the effects of thoracic gas compression on the maximal expiratory flow–volume (MEFV) curve without the use of a body plethysmograph. (**B**) One exercise tidal expiratory flow–volume curve plotted within two MEFV curves: (1) baseline before exercise (solid line), and (2) immediately after cessation of exercise (dashed line). Note the increase in maximal expiratory flow at all lung volumes in the MEFV curve obtained immediately after exercise. The tidal exercise breath was flow-limited when compared with the baseline MEFV curve but not the post-exercise MEFV curve. Failure to account for this exercise bronchodilation leads to overestimation of the presence and magnitude of expiratory flow limitation. The data are from a 28-yr-old adult male with asthma and moderate airway narrowing. Panel A reproduced with permission from [34] Panel B from authors’ laboratory.

To overcome the logistical challenges associated with the measurement of iso-volume pressure–flow curves, Hyatt et al. [29] focused on measures of expiratory flow and volume and developed a method to characterize the maximum capacity for expiratory flow across the range of lung volume from total lung capacity to residual volume during forced expiration [30]. When combined with measures of flow and volume during tidal breathing, assessments of EFL can be performed with relative ease. In this technique, the MEFV curve is derived from a forced vital capacity maneuver, and a TEFV curve is placed within the MEFV curve based on the volume obtained from an inspiratory capacity (IC) maneuver [31]. The occurrence of EFL is defined based on the overlap, or lack thereof, between the MEFV and TEFV curves, as shown in Figure 2A. Given its relative simplicity, this method has become ubiquitous and is the most common method of assessing EFL in both research and clinical settings. However, the method has well-documented limitations [32]. Participant cooperation is critical, and measurement errors may occur due to thoracic gas compression (Figure 3A), exercise-induced changes in bronchial tone (Figure 3B), incorrect alignment of TEFV curves, as well as differences in respiratory mechanical parameters between the TEFV and MEFV curves. The impact of these limitations is underscored by the observation that the repeatability of EFL measures obtained using this technique in healthy adults is poor [33], which emphasizes the need for novel methods of EFL assessment.

The method of overlaying TEFV and MEFV curves requires several measures, including an IC maneuver. Any error in the measurement of IC volume (and thus EELV), which can occur due to submaximal effort from the participant, will directly impact the validity of the measurement of EFL. Furthermore, this method only allows for the assessment of EFL in a relatively short time period immediately preceding the IC maneuver. Thus, an alternative method to assess EFL using flow–volume curves was developed [34]. The negative expiratory pressure technique involves the application of a negative pressure (i.e., ~5–15 cmH_2_O) at the mouth during a single expiration. The TEFV curve from this breath is then overlayed on the TEFV curve from the preceding expiration. The occurrence of EFL can then be determined based on the overlap, or lack thereof, between the two TEFV curves. This technique overcomes many of the limitations associated with overlaying TEFV and MEFV curves to assess EFL; it does not rely on participant cooperation, does not require forced expiratory maneuvers, and it can be performed in the absence of an IC maneuver. Nevertheless, the negative expiratory pressure technique can cause an involuntary collapse of the upper airway [35], a reflexive inhibition of expiratory muscle activity [36], and cannot be applied on a breath-by-breath basis. It also requires additional equipment to generate a negative mouth pressure, which further limits its application outside of research settings.

### 2.3. What Are the Physiological Consequences of Exercise EFL?

From a physiological standpoint, when EFL occurs, there are considerable negative impacts on the integrative responses to exercise, affecting the respiratory and cardiovascular systems, and impacting skeletal muscle function. First and foremost, the attainment of a plateau in the relationship between transpulmonary pressure and expiratory flow implies that some fraction of the pressure generated by the expiratory muscles is effectively ‘wasted’. Indeed, in healthy adults, EFL increases the expiratory component of the work of breathing and the metabolic cost of breathing [37]. In theory, an awareness of this increased expiratory muscle work may heighten the perception of dyspnea. Support for this contention comes from studies involving externally imposed EFL during exercise, whereby the perception of exertional dyspnea is greater in healthy males when EFL is imposed compared to control [38]. The presence of EFL can also lead to relative hypoventilation, and if exercise intensity is maintained, arterial hypoxemia may ensue. Relieving exercise-induced EFL experimentally using a normoxic helium–oxygen inspirate abolishes EFL, increases minute ventilation, and reverses hypoxemia in some individuals [39,40]. In some cases, EFL provokes an upward shift in operating lung volumes, which increases the ability to generate expired flow, thereby eliminating the presence of EFL [41]. If the increase in end-expiratory lung volume induced by EFL is sufficiently great, a phenomenon known as dynamic hyperinflation, the corresponding avoidance of EFL comes at a considerable mechanical and perceptual cost. The upward shift in operating lung volume means that breathing occurs along the less-compliant portion of the respiratory system’s pressure–volume relationship, which increases the elastic load on the respiratory muscles and the perception of dyspnea [42]. The high intrathoracic pressure swings associated with EFL have also been shown to alter the hemodynamic response to exercise [4,43]. Externally imposed EFL amplifies the effect of the respiratory muscle pump, causing a redistribution of blood volume towards the trunk [43]. Additionally, externally imposed EFL acutely reduces systemic oxygen delivery [4]. While the physiological impact of these observations is not yet known, it is reasonable to speculate that the hemodynamic effects of EFL during exercise may contribute to reducing exercise tolerance. In fact, externally imposed EFL has been shown to reduce exercise performance in healthy males compared to control [37]. Overall, the physiological consequences of EFL are considerable, affecting multiple organ systems and potentially impairing exercise tolerance. Collectively, these findings emphasize the importance of EFL as an outcome measure in the context of CPET.

## 3. Geometric Methods for Identifying EFL

Considering the limitations of the conventional method for measuring EFL, several other approaches for its identification that are not dependent on subject cooperation and the MEFV curve have been developed. Rather, the configuration of the TEFV curve itself is thought to reflect the prevailing mechanical events in the airways. And by extension, the shape of the TEFV curve should be responsive to the airway mechanical events existing under flow-limited conditions.

### 3.1. Shape-Based Measurements of TEFV Curves at Rest

**Figure 4.**
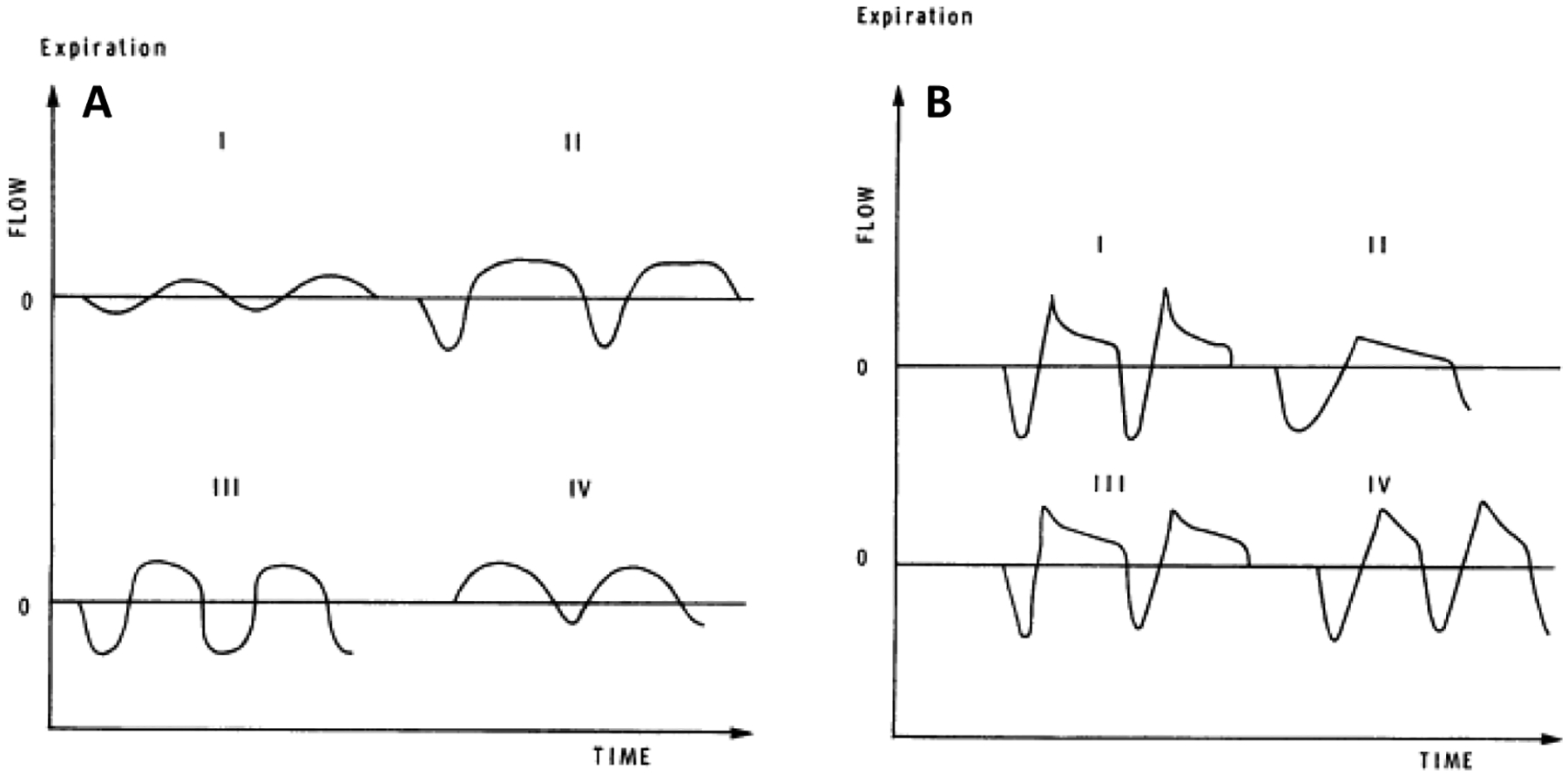
(**A**) Spontaneous resting tidal airflow in three healthy adults (I, II, III) and in one patient with restrictive lung disease (IV). (**B**) Spontaneous resting tidal airflow in four adults with obstructive airways disease. Reproduced with permission from [44]

Several investigators have developed quantitative measures that are sensitive to differences in resting, eupneic TEFV curve geometry. These analyses of resting breathing provide insight into the measurement and interpretation of exercise EFL. In 1981, Morris and Lane showed that the resting tidal expiratory flow pattern was different in healthy persons and in patients with obstructive lung disease [44]. In healthy adults in whom resting expiration is not flow-limited, the TEFV curve assumes a sinusoidal appearance where peak expiratory flow (PEF) occurs between 30% and 50% of the tidal volume (Figure 4A). By contrast, in patients with obstructive lung disease, PEF is reached earlier during expiration (10–30% of tidal volume) and assumes one of several phenotypes characterized by an elongated descending airflow that clearly deviates from a sinusoidal pattern (Figure 4B). These observations were quantified as the ratio between the volume expired before PEF and the total volume expired during the breath (ΔV/V%). In 1997, Baydur and Milic-Emili used the negative expiratory pressure technique to provide unequivocal evidence that mechanical EFL does occur during resting breathing in patients with moderate-to-severe COPD [45]. Moreover, conceptually similar measurements of resting TEFV curve geometry have yielded similar results, indicating that progressively worsened airway obstruction causes qualitative and quantitative changes to the TEFV curve [46–48].

### 3.2. Shape-Based Measurements of TEFV Curves During Exercise

**Figure 5.**
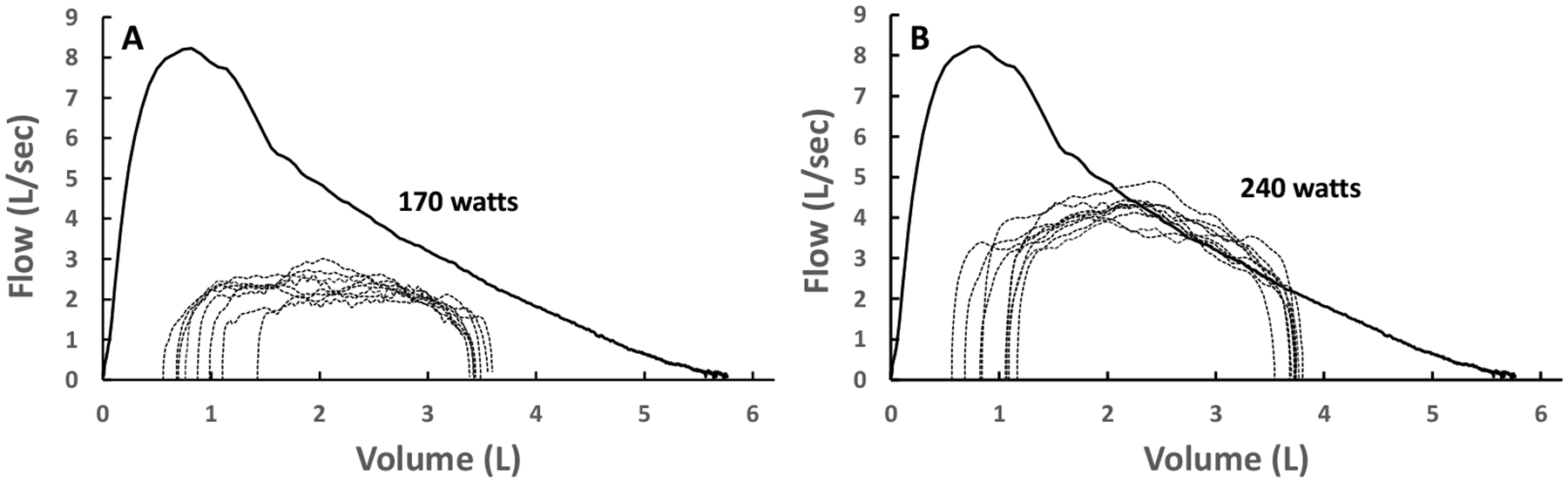
(**A**) Spontaneous tidal expiratory flow–volume (TEFV) curves during moderate exercise overlayed with the baseline maximal expiratory flow–volume (MEFV) curve in a healthy young male. Expiratory flow limitation (EFL) is not present. (**B**) Spontaneous TEFV curves during heavy exercise overlayed with the baseline MEFV curve in the same subject. In these breaths that exhibit EFL, the shape of the TEFV curves is similar to the shape and trajectory of the MEFV curve. Adapted from [50

Qualitative analyses have shown that flow-limited expirations (measured by the conventional method) often approximate the shape of the MEFV curve [17,49,50]. Figure 5A,B shows two sets of exercise TEFV curves plotted within the MEFV curve in a 21-year-old male with asthma and mildly narrowed airways. The breaths in Figure 5A are not expiratory flow-limited whereas the breaths in Figure 5B (higher exercise workload) do overlap with the MEFV curve. Qualitatively, the flow-limited TEFV curves appear to parallel the MEFV curve, beginning at the points of intersection. The general interpretation of these findings is that the trajectory of the spontaneous exercise TEFV curve essentially parallels that of the MEFV curve under conditions where maximal effective pressures—and maximal expiratory airflow—are approached or reached. This phenomenon is also nicely captured during graded expiratory efforts begun at total lung capacity (Figure 3A). Altogether, findings support the notion that EFL during exercise results in a TEFV curve where the expiratory limb takes on the appearance of the MEFV curve.

The rectangular area ratio (RAR) is a quantitative approach to identify EFL that is based on the shape of the TEFV curve [51]. In the analysis, a diagonal line connecting the point of maximum expiratory flow with the beginning of inspiration forms the diagonal of a rectangle. The RAR compares the area within the rectangle below the TEFV curve with the total area of the rectangle: RAR values greater than 0.5 indicate that the expiratory limb is linear or convex, whereas RAR values <0.5 indicate TEFV curves that are concave. Two studies quantified the RAR during exercise in patients with obstructive lung disease and in healthy controls [51,52]. In both reports, TEFV curves were convex in healthy control subjects and the RAR generally increased during exercise. In contrast, both resting and exercise TEFV curves were concave in obstructive lung disease patients, becoming progressively more concave during exercise of increasing intensity; RAR progressively decreased during exercise. The major strength of this method for determining EFL is that it does not require an MEFV curve. However, in healthy persons or in persons with mild obstructive lung disease or restrictive lung disease, the expiratory limb of the TEFV curve need not be concave under conditions of EFL; it remains linear or convex. Thus, the RAR is not a useful method for identifying EFL in such persons. For instance, in patients with interstitial lung disease, the exercise TEFV curve is linear or convex even when EFL is present [53]. The fact that the RAR will not be able to identify EFL in situations where the TEFV curve is not concave greatly limits its potential utility.

Recently, Welch and colleagues developed a quantitative, vector-based analysis that compares the configuration of the TEFV curve with the MEFV curve [54]. The analysis generates multiple lines (vectors) connecting points on the TEFV curve with the MEFV curve and then applies trigonometry to quantify conformity of the tidal expiration with the slope and shape of the MEFV curve. Breaths that parallel and conform to the trajectory of the MEFV curve are deemed expiratory-flow-limited. In 25 healthy adults and 20 patients with obstructive lung disease, the identification and severity of EFL were similar using the vector-based method and the conventional method. Similar to the RAR, the advantage of this method is that it is based on the configuration of the TEFV curve itself and not on the overlap of the tidal with the MEFV curve. Thus, the position of the TEFV curve within the MEFV curve is immaterial to the analysis. The method would presumably identify EFL in cases where it was present, but a poor inspiratory capacity maneuver overestimated operational lung volumes. As well, in cases where the TEFV curve meets or surpasses the MEFV curve, EFL will only be identified if the two expiratory limbs have the same conformation. Nevertheless, this approach still depends on the MEFV curve, requiring patient effort and cooperation, and the confounding effects of thoracic gas compression and exercise bronchodilation.

### 3.3. Deep Machine Learning to Identify EFL

In recent years, advancements in computing power and data availability have allowed for increased access and improved ability to apply artificial intelligence towards a variety of biomedical questions and problems [55]. Deep learning is one application of artificial intelligence with particular promise as a tool for addressing relevant problems in medical practice, including identification, classification, and treatment of pulmonary disease [56,57]. Deep learning designs artificial neural network (ANN) models to process and analyze data. The overall structure of an ANN is based on neuronal communication in humans, where various neurotransmitters released by first-order neurons bind with receptors located on second−order neurons. However, in an ANN, simple mathematical functions act as the “neurotransmitters” that communicate information from a first- to a second-order neuron. Artificial neural networks are “trained” on large amounts of data to predict data classifications in response to one or more input variables; the predictive ability of an ANN improves—learns—as data are run through the algorithm. A convolutional neural network (CNN) is a type of ANN that is particularly adept at pattern recognition and detecting shapes and boundaries in time series data. In fact, TEFV curves are plotted and analyzed as a function of either time or volume during expiration. Given the temporal nature of the TEFV curve and that it consists of a closed, two-dimensional shape with a clear boundary, an appropriately constructed CNN might be able to discern differences in overall shape in TEFV curves that are flow-limited vs. those that are not.

**Figure 6.**
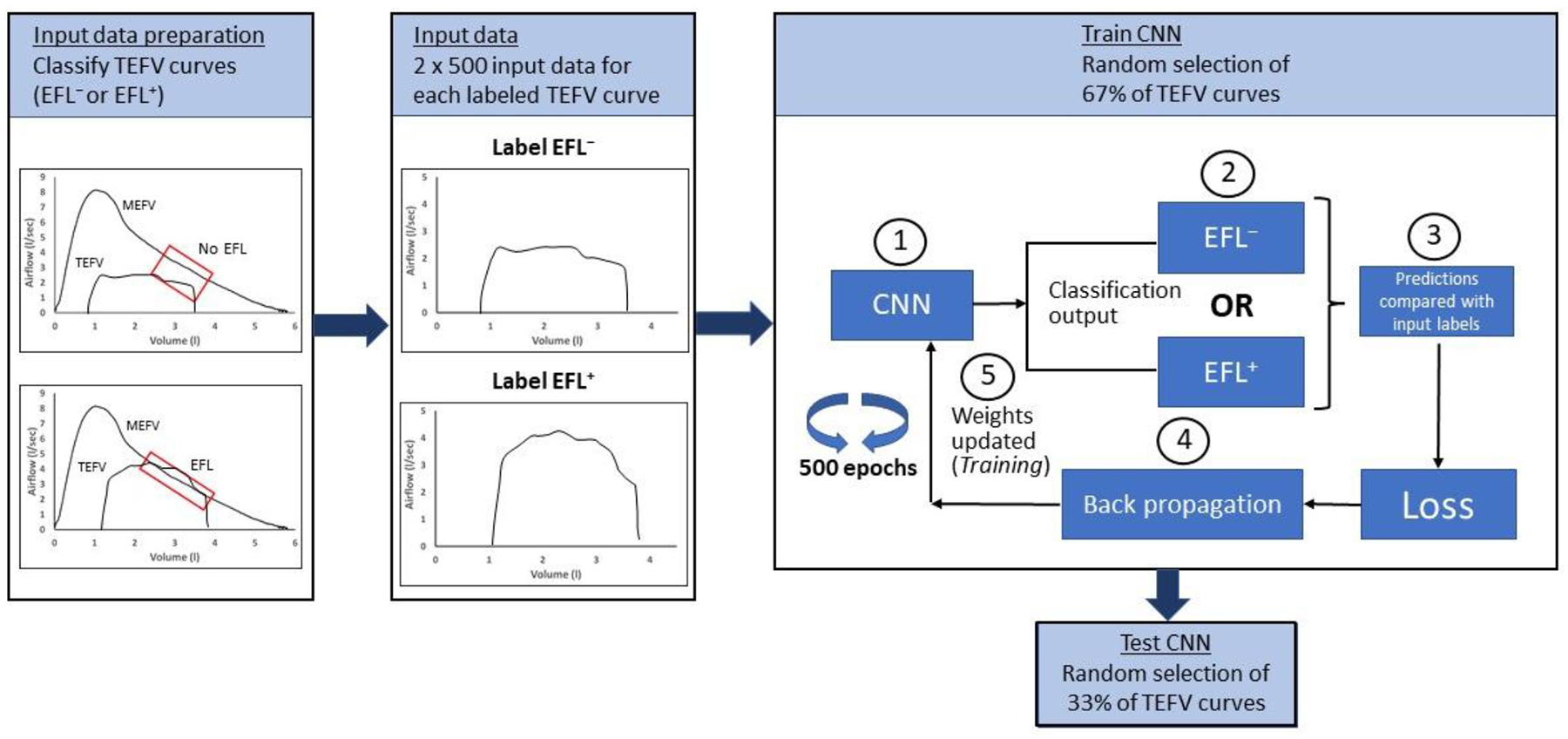
Schematic illustrating overall steps in application of a neural network to identify exercise expiratory flow limitation. Data preparation: All TEFV curves from all subjects in the dataset are overlayed with each subject’s MEFV curve. The conventional method for determining EFL is used to identify all TEFV curves as EFL^+^ or EFL^−^. Input data: All labeled TEFV curves are prepared for input into the CNN by resampling such that all curves are in a 2 × 500 format. Note that this input format is not obligatory; rather, the format for input data is determined by the investigators after considering the strengths and limitations of the possible formats and the level of detail needed to discriminate between the labeled data. Train CNN: A random selection process selects 67% of the TEFV curves for the training. (1) All labeled, randomly selected TEFV curves are entered into the CNN, (2) forward passed through the CNN and categorized as EFL^−^ or EFL^+^, (3) the CNN categorization is compared with the label to calculate loss (i.e., difference between the input label and the CNN’s categorization), (4) the error is propagated backwards (backpropagation) through the CNN to (5) update the weights in a way that minimizes the algorithm’s error, potentially improving accuracy, (6) the training data are passed through the CNN 500 times (epochs). Test CNN. TEFV, tidal expiratory flow–volume; MEFV, maximal expiratory flow–volume; EFL, expiratory flow limitation; CNN, convolutional neural network.

We recently developed a CNN to identify exercise EFL in TEFV curves from *n* = 22 adults in whom baseline airway function ranged from above-predicted values to mildly obstructed [50]. All 22 subjects completed an incremental exercise test, and a total of 2931 TEFV curves from the tests were placed within the pre-exercise MEFV curve and labeled as “non-EFL” or “EFL” using the conventional method. After building the CNN and adjusting its parameters to attain maximum performance, the final CNN achieved an accuracy of 90% at correctly categorizing the TEFV curves as flow-limited or not. Furthermore, the CNN was equally effective at categorizing TEFV curves in subjects with normal spirometry and in those with mild airway obstruction (FEV_1_/FVC between 0.6–0.7). While a detailed description of the concepts, features, and processes involved in CNNs is well beyond the scope of this review, Figure 6 illustrates the overall approach and the most important features of the CNN.

This novel method of identifying exercise EFL is promising. The CNN neither requires an MEFV curve nor that operational lung volumes be determined; thus, patients do not need to perform inspiratory capacity maneuvers during the exercise. Furthermore, since the configuration of the TEFV curve should be responsive to EFL in both healthy persons and in persons with impaired lung function, the CNN ought to be equally effective in all populations. In theory, a practitioner could simply select a series of exercise breaths, input them into a CNN with demonstrated ability to differentiate breaths that are, and are not, expiratory flow-limited, and provide a very good idea of the presence of EFL with exercise.

**Figure 7.**
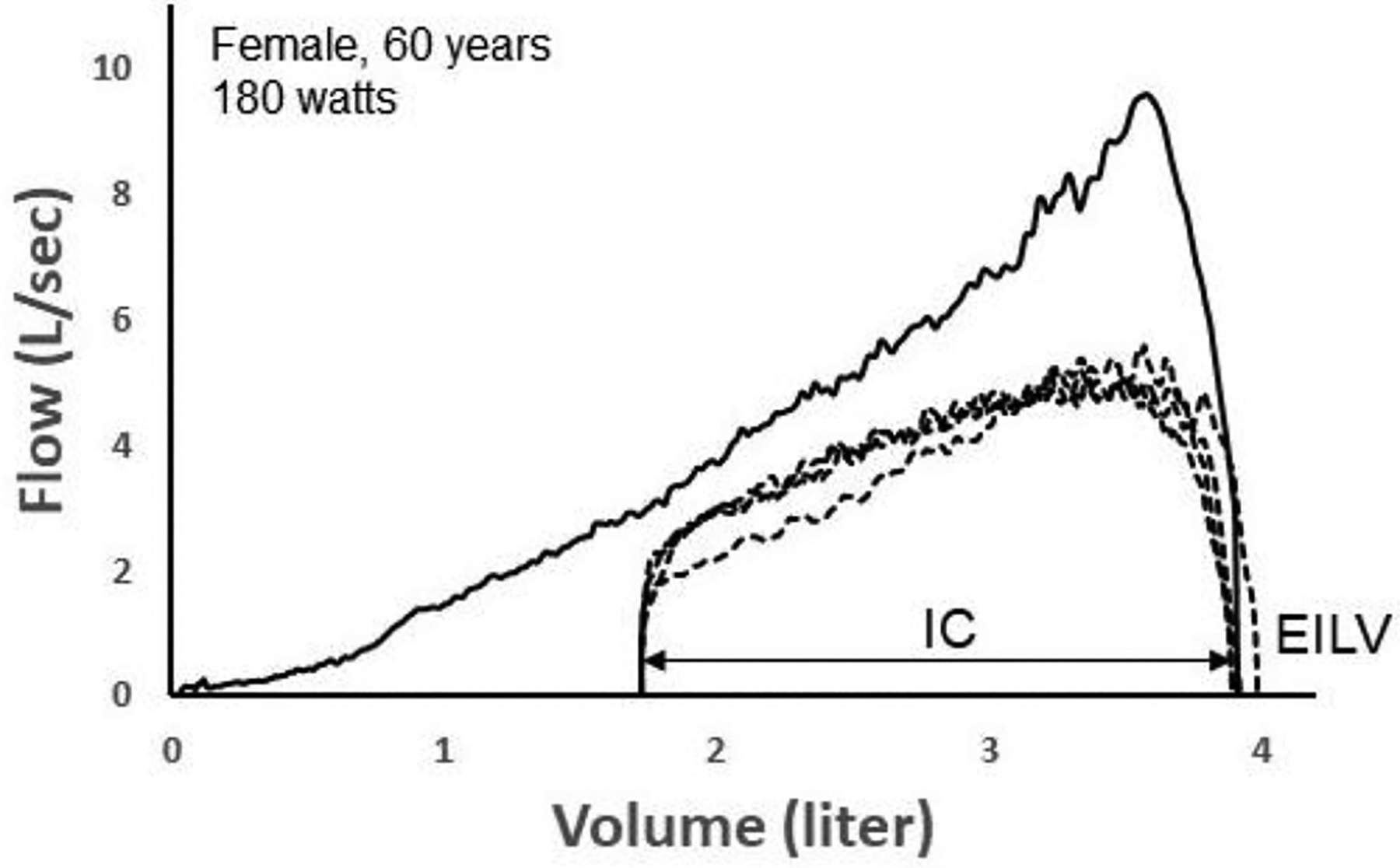
A maximal expiratory flow–volume (MEFV) curve (solid line) with four tidal expiratory flow–volume (TEFV) curves (dashed lines) during exercise in a 60-yr-old female. Since the TEFV curves do not meet the MEFV curve, the four breaths were labeled as EFL^−^ (non-expiratory flow-limited). However, the CNN categorized each breath as EFL^+^. The shape of the TEFV curves is suggestive of EFL. Also, the unreasonably high end-inspiratory lung volumes (EILV) suggest that the inspiratory capacity (IC) was not performed properly, thus, mistakenly overestimating operational lung volume and making it appear that EFL was not present. Data from authors’ laboratory.

Here, we provide two informative examples that demonstrate the promise of deep learning to identify exercise ventilatory constraint. Figure 7 depicts four TEFV curves plotted within a pre-exercise MEFV curve in a 60 yr female during exercise on a cycle ergometer. Exercise end-inspiratory lung volume (EILV) was unreasonably high, equaling or exceeding TLC. This suggests that the inspiratory capacity maneuver was not performed properly. Since the TEFV curves do not meet the MEFV curve, the exercise breaths were entered into the CNN labeled as “non-EFL.” However, following analysis by the CNN algorithm, it categorized all four TEFV curves as EFL^+^. In this case, the four breaths would thus be categorized as “false positives” since they were categorized by the CNN as EFL^+^ despite being labeled as non-EFL. However, we think it is likely that the breaths were, in reality, flow-limited. The configuration of the TEFV curves in combination with the very high EILV does suggest that EFL was present. Indeed, a reduction in operating lung volume of only 0.25 L (i.e., 0.25 L increase in the inspiratory capacity) would result in overlap of the TEFV curves with the MEFV curve. In effect, the four breaths might be more appropriately categorized as *false negatives* according to the conventional method.

**Figure 8.**
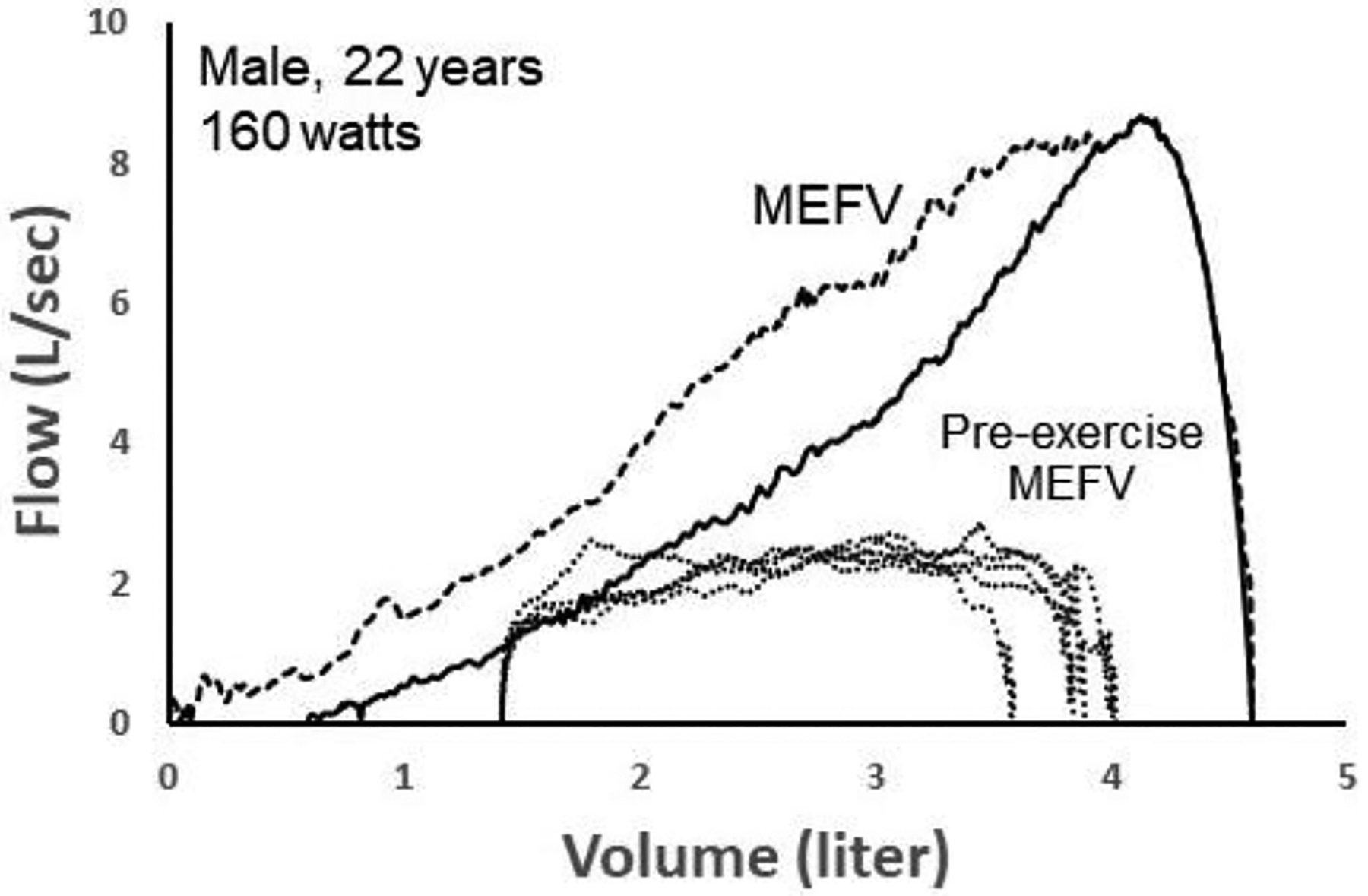
Two maximal expiratory flow–volume (MEFV) curves with five tidal expiratory flow–volume (TEFV) curves (dotted lines) during exercise in a 22-year-old male. The solid MEFV curve was obtained as a single maneuver before exercise (Pre-exercise MEFV), whereas the dashed MEFV curve represents the highest airflow at all lung volumes after accounting for thoracic gas compression and exercise-induced bronchodilation. Since the TEFV curves intersected the pre-exercise MEFV curve, they were labelled as expiratory flow-limited (EFL^+^) and input into the convolutional neural network (CNN). However, the CNN classified all four TEFV curves as EFL^−^. Data from authors’ laboratory.

Figure 8 shows five spontaneous TEFV curves plotted within two MEFV curves in a 22 yr male exercising at 160 watts on a cycle ergometer. The green MEFV curve (pre-exercise MEFV) was performed as a single maneuver before exercise. The larger, blue MEFV curve (MEFV) was constructed by plotting the highest expiratory airflow achieved at all lung volumes during a series of graded expiratory efforts before and after exercise, minimizing the influence of thoracic gas compression and accounting for exercise bronchodilation [58]. As shown, EFL was present when the TEFV curves were overlayed with the pre-exercise MEFV curve, whereas it was not present when compared with the larger MEFV curve. Whereas the five breaths were labeled as EFL^+^ (determined by the pre-exercise MEFV), the CNN algorithm categorized them as non-expiratory flow-limited. In this example, it is likely the case that the breaths were not, in reality, flow-limited, since expired airflow did not meet the larger MEFV curve that more accurately reflects actual airway caliber during the exercise. Similar to the previous case shown in Figure 7, these findings suggest that the CNN’s ability to discern predominant airway mechanical events based on the shape of the spontaneous TEFV curve might be more accurate than the conventional method of identifying EFL.

The field is in the beginning stages of exploring the ability of deep learning to accurately identify ventilatory constraints during exercise. While our initial studies are promising, much work must be done before this approach can conceivably be integrated into clinical practice. Foremost, the technology must be tested, and shown to be effective, in the various patient populations (e.g., COPD, heart failure) most likely to benefit from improved methods for identifying ventilatory constraints. Finally, none of the methods for identifying exercise EFL are flawless, and they all require some amount of nuance. None of the approaches will ever be able to measure EFL with 100% accuracy.

### 3.4. Summary

Cardiopulmonary exercise testing is a uniquely effective approach for uncovering functional maladaptations to multiple organ systems and physiological processes. Exercise testing with continuous measurement of airflow is particularly effective at identifying causes of unexplained shortness of breath, exaggerated exertional breathlessness, and ventilatory constraints impacting exercise tolerance. Expiratory flow limitation is one indicator of ventilatory mechanical constraint that increases ventilatory neuromuscular output without accompanying increases in expired airflow. Inevitably, this uncoupling between ventilatory effort and outcome negatively contributes to the perceptual responses to exercise and exercise capacity. For several reasons, EFL is not usually assessed during CPET, representing a significant lost opportunity for maximum insights. A primary factor limiting the ability to measure EFL is that its measurement has traditionally required comparison of the exercise tidal breaths with the MEFV curve (a voluntary maneuver with several limitations) and performance of IC maneuvers by the patient. This review provided a discussion of EFL, including its physiology, approaches for its measurement during exercise and the limitations of each approach, and the physiological consequences of EFL. Because of the intractability of the limitations of current methods for assessing exercise EFL, novel approaches for its measurement need to be examined. We discussed the potential of shape-based analyses of the exercise spontaneous TEFV curve to identify EFL that have several advantages over the conventional methods for its measurement. The final section of this review describes recent findings highlighting the exciting potential of modern deep learning technology for identifying exercise EFL based on the shape of the spontaneous TEFV curve.

## Data Availability

The data presented in figure 7 and figure 8 are available on request from the corresponding author.
